# Improvement in staff behavior during surgical procedures to prevent post-operative complications (ARIBO^2^): study protocol for a cluster randomised trial

**DOI:** 10.1186/s13063-019-3370-z

**Published:** 2019-05-20

**Authors:** Gabriel Birgand, Thomas Haudebourg, Leslie Grammatico-Guillon, Léa Ferrand, Leila Moret, François Gouin, Nicolas Mauduit, Christophe Leux, Yannick Le Manach, Didier Lepelletier, Elsa Tavernier, Jean-Christophe Lucet, Bruno Giraudeau

**Affiliations:** 1 CPias Pays de la Loire, Nantes University Hospital, CHU – Le Tourville, 5, rue du Pr Yves Boquien, 44093 Nantes, cedex France; 2 Health Protection Research Unit, Imperial College London, London, UK; 3 Service de Santé Publique, Unité Régionale d’épidémiologie Hospitalière, CHU, Université de Tours, Tours, France; 4 Direction de la Recherche Clinique, Nantes University Hospital, Nantes, France; 5 Service de Santé Publique, Nantes University Hospital, Nantes, France; 6 Service de Chirurgie Orthopédique, Nantes University Hospital, Nantes, France; 7 Service D’information Médicale, Nantes University Hospital, Nantes, France; 8 Perioperative Medicine and Surgical Research Unit, Population Health Research Institute, David Braley Cardiac, Vascular and Stroke Research Institute, 237 Barton St E, Hamilton, ON L8L 2X2 Canada; 9 Unité de Gestion du Risque Infectieux, CHU, Nantes, France; 10 INSERM CIC 1415, CHRU de Tours, Tours, France; 11 Université de Tours, Université de Nantes, INSERM SPHERE U1246, Tours, France; 12 Unité d’hygiène et de lutte Contre L’infection Nosocomiale (UHLIN), AP-HP, Paris, France

**Keywords:** Surgery, Complications, Surgical site infection, Behaviour, Noise, Doors opening

## Abstract

**Background:**

Inappropriate staff behaviour during surgical procedures may disrupt the surgical performance and compromise patient safety. We developed an innovative monitoring and feedback system combined with an adaptive approach to optimise staff behaviour intraoperatively and prevent post-operative complications (POC) in orthopaedic surgery.

**Methods/design:**

This protocol describes a parallel-group, cluster randomised, controlled trial with orthopaedic centre as the unit of randomisation. The intervention period will last 6 months and will be based on the monitoring of two surrogates of staff behaviour: the frequency of doors opening and the level of noise. Both will be collected from incision to wound closure, using wireless sensors and sonometers, and recorded and analysed on a dedicated platform (Livepulse®). Staff from centres randomised to the intervention arm will be informed in real time on their own data through an interactive dashboard available in each operating room (OR), and *a posteriori* for hip and knee replacement POC. Aggregated data from all centres will also be displayed for benchmarking. A *lean* method will be applied in each centre by a local multidisciplinary team to analyse baseline situations, determine the target condition, analyse the root cause(s), and take countermeasures. The education and awareness of participants on the impact of their behaviour on patient safety will assist the quality improvement process. The control centres will be blinded to monitoring data and quality improvement approaches. The primary outcome will be any POC occurring during the 30 days post operation. We will evaluate this outcome using local and national routinely collected data from hospital discharge and disease databases. Thirty orthopaedic centres will be randomised for a total of 9945 hip and knee replacement surgical procedures.

**Discussion:**

The field of human factors and behaviour in the OR seems to offer potential room for improvement. An intervention providing goal-setting, monitoring, feedback and action planning may reduce the traffic flow and interruptions/distractions of the surgical team during procedures, preventing subsequent POCs. The results of this trial will provide important data on the impact of OR staff behaviour on patient safety, and promote best practice during surgical procedures.

**Trial registration:**

ClinicalTrials.gov, NCT03158181.

**Electronic supplementary material:**

The online version of this article (10.1186/s13063-019-3370-z) contains supplementary material, which is available to authorized users.

## Background

Approximately 234 million surgical procedures are performed each year worldwide [1]. In France, hip and knee replacements generated more than 190,000 procedures in 2015 [2]. The operating room (OR) appears to be the main place of severe adverse events in hospitals [3, 4]. Approximately 14.4% of patients report an adverse event during their surgical care, among which 3.6% lead to a fatal outcome [5]. The rate of adverse post-operative outcomes (complications, readmissions and mortality) following total hip replacement has been estimated at 9% in France [6], making orthopaedic surgery an important target for national prevention programmes [7].

The performance and quality of surgical care depends on the technical and non-technical skills of the surgical team [8]. Traditional approaches to surgical safety focusing on technical aspects have led to a decrease in post-operative complications (POC). For instance, infection control measures have contributed to a reduction by half in the rates of surgical site infection (SSI) during the past two decades [9]. Despite obvious improvements, the rates of POC have remained stable for several years. The rates of some complications, including SSI following total hip replacement, are even rising in France [10].

The field of human factors and behaviour in the OR seems to represent potential room for improvement [11]. The OR is a highly specialised environment where sophisticated techniques are used, generating many risks for patients [12]. Surgical teams are subject to high stress levels and to special rules, leading to special sociological characteristics [13]. In consequence, myths and rituals abound in surgery, sometimes with little reliance on evidence to inform practice in the OR [14]. Intraoperative behaviour has been described by other authors as “sacred cows” or “ritualistic practice” [15, 16].

The atmosphere within the workplace of professionals has a direct influence on the quality of care, with immediate post-operative consequences in the case of failure [17]. This factor depends on the safety culture and the organization in the OR. Team work, communication, leadership and management by leaders are major determinants of the work climate. Local context and culture, characteristics of teams, along with local systems impact on frontline practice in the OR [18]. A change in existing norms and practices may be difficult to obtain [19]. Despite the specificity of this environment, OR staff practices may become routine, and staff may sometimes omit basic rules of discipline for patient safety.

Several guidelines refer to specific types of behaviour as potential risk factors for POC [20, 21]. National recommendations emphasize the importance of discipline during surgical procedures [22]. However, these recommendations are often vague, based on expert recommendations, without robust scientific evidence. The current literature suggests high variation in door-opening events during surgery (among which one third are considered avoidable) [23]. Opening doors may lead to airborne contamination and a subsequent increased risk of SSI [24, 25]. This risk is major during surgery involving a cutaneous approach, and belonging to the clean contamination class (Altemeier’s class I), such as orthopaedic or cardiac surgery. These data were recently confirmed by a multicentre observational study performed in France using new technological tools, describing correlation between entries/exits in the OR (median 29/h in orthopaedic surgery, minimum–maximum 16–65), staff movements and air particle counts [11]. The atmosphere in the OR seems to also be critical for patient safety. A noisy environment during procedures has consequences on patient comfort, anaesthesia and surgical practices [26, 27]. Hence, a high level of noise is associated with the occurrence of intra-operative and post-operative adverse events including SSI [28–30]. Initiatives to reduce the level of noise during procedures have led to improvement in patient safety [31]. However, to date, no interventional study using an adequately robust method (randomised controlled trial) has demonstrated the impact of improvements in intra-operative behaviour on the prevention of POC, including SSI.

### Aims and objectives

The main objective is to assess the impact of a bundle of measures optimising discipline (frequency of door opening and level of noise) in the OR in preventing POC in prosthetic hip and knee orthopaedic surgery. Secondary objectives are to assess (1) the impact of a bundle of measures to optimise OR discipline for each element of POC composite criteria; (2) the impact of a bundle of measures to optimise the traffic flow in the OR using process indicators (number of door openings, level of noise and interruptions/distractions) of surgical team behaviour during procedures; and (3) the sustainability of the approach in a 6-month post-intervention follow up.

### Trial design

**Fig. 1:**
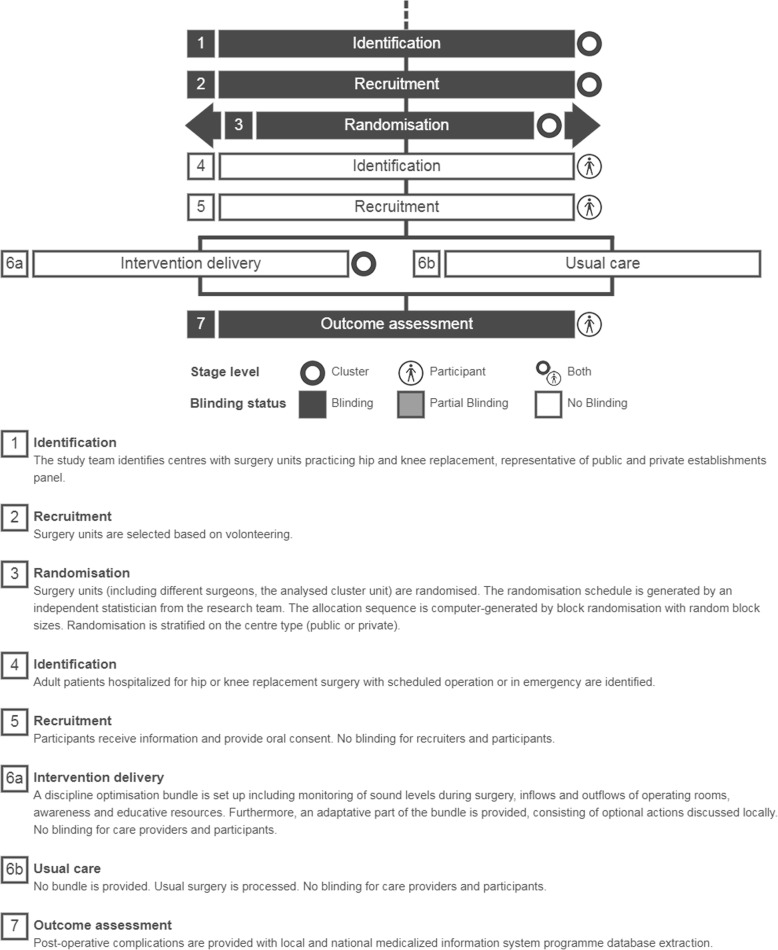
Timeline cluster: graphical tool to identify risk of bias in cluster randomised trials [47]

This parallel-group, cluster randomised, controlled trial is designed to evaluate the superiority of a multimodal intervention (monitoring and feedback of behavioural data combined with an adaptive improvement approach) compared with routine practice. The hospital will be the randomisation unit. Cluster randomisation will be used because of the non-individual level of the intervention. Indeed, the intervention applies at the surgical-team level based on group behaviour. An individual randomisation would have been a source of group contamination. Although the intervention applies at the surgical-team level, randomising surgeons was not desirable since other team members, who work with different surgeons, would have also been a source of contamination. In the end, randomisation will be performed at the hospital level, but the surgeon will be the unit of clustering in the statistical analysis as described below. We used the Standard Protocol Items Recommendations for Interventional Trials (SPIRIT) Checklist to guide the reporting of our protocol [32] and the Template for Intervention Description and Replication (TIDieR) to guide the reporting of components of our intervention [33] (Fig. 1).

## Methods and analysis

### Participants, interventions and outcomes

#### Study setting

We will conduct the trial in a selection of 30 orthopaedic surgical centres delivering knee or hip arthroplasty. The study will take place in western France and the Paris area. Centres performing more than 200 knee/hip arthroplasty per year will be contacted via local infection control and health quality teams. This group of facilities will provide a representative panel of public/private settings with variable sizes and activities. The ORs from selected centres can be equipped with or without laminar airflow, and without restriction of surface or number of doors. Centres involved in similar ongoing research on behaviour change in the OR will be excluded.

#### Patient eligibility criteria

The population will comprise patients ages 18 years or older undergoing the surgical procedure of knee or hip arthroplasty (replacement or resurfacing) as a primary, elective or urgent referral. Patients undergoing arthroplasty revision and patients with previous SSI will not be eligible. All surgical procedures will be included, whatever the age, gender, experience and position of the patient’s senior orthopaedic surgeon.

#### Intervention

The intervention involves two components: (1) a standardised system for the monitoring of and feedback on the frequency of doors opening and noise levels and (2) an adaptive approach aiming to reduce the frequency of doors opening and noise levels.

##### Component 1 – monitoring, feedback and benchmarking process

This component will be common to all centres within the intervention arm. Process indicators including the frequency of door opening per hour and the level of noise (mean level of noise measured in decibels and mean decibels (dB) above the baseline) will be collected through an innovative system. Door opening sensors will be placed on each door in every OR. A weather station sonometer will be placed next to the anaesthesia machine to continuously collect data on noise levels. All devices will be connected to a gateway for the wireless communication of data through a router (Wifi 3G) to a platform (Livepulse®). The Livepulse® platform analyses and displays the data. A dashboard available through a web application (for computers, tablets and smartphones) will be made available to all surgical staff involved in the intervention arm.

The direct-utilisation dashboard gives the opportunity to monitor the cumulative number times the doors are opened and the average level of noise in real time. The times of cutaneous incision and wound closure are not interfaced in real time by the system. To overcome this issue, the application allows start of the recording phase in the OR with direct feedback. Ideally, the beginning of the recording will correspond to the incision during the hip/knee arthroplasty. The number of times this direct feedback application is used by OR staff will be visible to the investigator. Reminders will be sent to the poorly compliant users and incentives will be given to frequent and assiduous users. The accessibility of this application will be facilitated by the absence of a password, its presence on the desktop background of the OR computers and its availability on tablet computers dedicated to the project or on personal smartphones.

Information on the procedure (type, date, time of incision/closure, senior surgeon, OR identification, patient identification number) will be downloaded weekly on a server developed for the project. These data will be merged with the behavioural data collected on the Livepulse® platform, to build a weekly dashboard focusing on door opening and noise levels from the time of incision to wound closure for all hip/knee replacements, globally, by centre and by OR. These dashboards will be displayed in the OR, discussed during weekly OR staff meetings and presented during hospital board meetings. Individual reports will also be sent to each senior surgeon.

Awareness and educational tools will also be part of the core measures. They will focus on the impact of OR staff behaviour on the risk of POC. This will be based on a 30-min presentation and discussion with staff in the OR at the beginning of the intervention phase. Sessions will be organised to cover all OR staff and new arrivals. A slide show including the impact of door opening and noise on the risk of POC will be presented, based on the data reported in the literature [24] and the experience acquired during the previous ARIBO study [11]. This presentation will also explain how to use the monitoring system, and detail the adaptive approach of the intervention. Communication tools have been created for the project including videos and animations showing the role of air ventilation systems and the impact of door opening on their efficacy. Posters and leaflets will be created to promote the absence of unnecessary door opening and respect of calm in the OR during procedures.

##### Component 2 – adaptive approach

This adaptive component will search for root causes of door opening and high noise levels, with the aim of building a tailored action plan. The causes of deviant behaviour and the countermeasures undertaken will be context-dependent. We will use the *lean* methods (methods of the Toyota Production System) based on a plan-do-check-act (PDCA), to reduce door opening and noise levels during surgery [23].

A multidisciplinary team will be created in each centre including orthopaedic surgeons, anaesthesiologists, surgical nurses and quality coordinators, who will be mentored by a lean coach (either infection control specialist or quality coordinator) during the entire intervention period. Each centre will designate a local project liaison and a local project champion. Project liaison will be an infection control/quality and safety specialist and the project champion a member of the OR staff (surgeon or nurse) with strong leadership skills. This adaptive method will be divided into 8 successive phases:

1. Clarifying the problem: the baseline data collected before the intervention period by the monitoring system in the ORs will set the context in each centre/OR and across centres/ORs.
2. Specifying the current situation: the reasons for door opening and assessment of communications, interruptions and distractions will be audited by members of the multidisciplinary team, including the local project liaison and champion, using validated observation forms [34, 35].
3. Multidisciplinary analysis of data collected during the audits: these data will be presented and discussed to illustrate the baseline situation. The following questions will be addressed by the multidisciplinary team: who is opening doors/distracting/interrupting and for what reason; what is the variability among senior surgeons; when do the events occur.
4. Determining the target condition: the multidisciplinary team will state the ideal conditions in term of door opening and noise levels (e.g. zero door opening between the time of the incision and closing of the wound, except for specific clinical reasons, namely a need for x-rays, unexpected materials, instruments or blood products; breaks or service shifts of employees; emergencies and/or supervision for the orthopaedist or anaesthetist).
5. Analysing the root cause(s): the multidisciplinary team will analyse the gap between the baseline and the target conditions using an Ishikawa diagram, also known as a fish-bone diagram or cause-and-effect diagram [20]. This diagram uses the major categories “people, machines, methods and materials” to analyse the reasons for events.
6. Action plan: taking countermeasures.
7. Evaluating the impact of the action plan using the monitoring system.

Table 1 provides a further overview of the intervention as per TIDieR criteria.

**Table 1:** Overview of the ARIBO^2^ intervention, as per Template for Intervention Description and Replication (TIDieR) criteria

TIDieR criteria	Description of intervention and quality control procedures
Brief name	ARIBO^2^ intervention
Why?	Inappropriate staff behaviour during surgical procedures may lead to a low performance and compromise patient safety. An innovative monitoring and feedback system combined with an adaptive approach will be developed and evaluated to optimise intra-operative staff behaviour and prevent post-operative complications (POC) in orthopaedic surgery
What materials?	An online application will provide data on door opening and level of noise in each participating operating room (OR) equipped with door sensors and sonometers, through a dashboard. Education material (slide shows) and communication tools (posters, leaflets and videos) delivered to OR staff
What procedures?	A core measure will be the monitoring, feedback and benchmarking of door opening and noise levels through the online dashboard. This will be added as an education and awareness process. An adaptive approach based on a lean method (plan-do-check-act) will provide a contextual analysis and tailored action plan to reduce door opening and optimise the level of noise during orthopaedic surgery. The intervention will be characterized by various steps: clarifying the problem, specifying the baseline situation, determining the target condition, analysing the root cause(s) and taking countermeasures
Who provided?	A local project liaison will be the link between the centre and the study team. She/he will participate in the implementation of the intervention (education and training, awareness, communication). The local project champion will be in charge of daily activities in the OR. A training session will be organised by the investigators for the project liaison and champion to give them key messages, detail the intervention and present the tools developed for the study Both will create an internal multidisciplinary group adapted for the design and implementation of the adaptive measures The multidisciplinary team will consist of an orthopaedic surgeon, an anaesthesiologist, a surgical nurse and a quality coordinator. They will be involved in the adaptive approach along the entire intervention period
How?	Online dashboard providing feedback to OR staff in real time on their intra-operative behaviour (door opening and noise) and weekly, specifically during hip and knee replacement
Where?	Orthopaedic operating rooms
When and how much?	The intervention will be available for 6 months. A given patientwill only be exposed to the intervention once (primary hip and knee replacements).
Tailoring	The adaptive approach will be tailored to the local context and current situation on door opening and noise in the OR. The multidisciplinary team will participate in the design of action plans tailored and adapted to the context

### Enhancing the fidelity of delivery and adherence to the intervention

This adaptive approach will be inspired by the Comprehensive Unit-based Safety Programme (CUSP) approach [36]. This approach is based on the overarching engagement of local leadership and the promotion of accountability of front-line staff and senior leaders. The intervention will be implemented entirely by local teams. The local project liaison will be the direct link between the centre and the study team. The local project champion will be in charge of daily activities in the operating suite. They will both participate in the implementation of the intervention (education and training, awareness, communication). They will mentor the internal multidisciplinary group in charge of the adaptive approach as described above. A preliminary workshop will be organised, gathering all liaisons/champions to present the project in details, with the expectations, roles and responsibilities in the project, and discuss the key elements of the intervention. The subsequent workshops will be held quarterly to identify any difficulties in implementation, find solutions collectively and discuss evolutions.

Local liaisons/champions will discuss actions and data with the surgical teams during monthly meetings. Project staff will form a central team providing technical expertise and mentorship on project management. This coordination will be delivered at distance through monthly webinars, e-mails, and telephone discussions, until the end of follow up. During the study, each site will receive one support visit from the project team, and will participate in two inter-site meetings. Between-site exchange of information will be encouraged throughout the study.

The engagement will be insured through dedicated senior management (head of orthopaedic surgery, anaesthesia and surgery committee acting as the project champion), physician and nursing leadership engagement for review and feedback on the action plan, writing and pledging commitment in a statement and endorsement of the action plan by all surgeons and anaesthetists. Regular and periodic meetings will be organised with all stakeholders engaged to identify expectations, brainstorm about causes and effects, plan and map implementation processes, review progress and measure outcomes.

Adherence to the intervention will be assessed by four different methods. First, data will be systematically collected during monthly teleconference and quarterly meetings. The initial data will include the starting date and the creation of a multidisciplinary team. Then, champions will be questioned on the internal communication performed, use of the monitoring system, the internal analysis of behavioural data collected by the monitoring system, audits performed, improvement actions (countermeasures) designed and implemented and good and bad points observed. Second, an external assessment of the engagement of champions and centres will be regularly performed by two different research team members (TH and GB). Both will assign a score on a Likert scale (with a score of 1 meaning poor engagement and a score of 5 meaning total adherence) at each step of the study process, including fluidity of contacts, organisation of kick-off meetings, set-up of the material in the OR, general answers to questions and overall reactivity. The study team will quantify the number of connections to the monitoring application by the centres, with the possibility to distinguish the pages accessed. Finally, a qualitative study with semi-structured interviews is planned at the end of the study to assess barriers and facilitators of the implementation process.

### Control conditions

As for the intervention arm, the monitoring system will be installed in the 15 orthopaedic centres participating in the control arm. Thus, data on door opening and noise levels will be collected for every hip/knee arthroplasty. However, these centres will be blinded to the data, the application and the dashboard. Intervention materials and training (e.g. slide shows, videos, posters), and the methodology of the adaptive approach will not be provided.

### Outcomes

The primary outcome is the occurrence of any major POC (i.e. a composite of SSI, thromboembolic complications, unplanned surgical revision, in-hospital mortality, myocardial infarction, cardiac failure, stroke, renal failure/dialysis and sepsis) (Table 2) during the postoperative period, up to 30 days after the procedure (90 days for SSI). We will also evaluate readmission for any cause within 72 h after initial hospital discharge, ICU admission and in-hospital mortality during readmission. Secondary outcomes will include each of the individual components of the composite described above [37]. The registry-based design uses routinely collected data to ascertain our outcomes. In France, we have databases of routinely collected data on hospital discharge (acronym PMSI, hospital billing records) and *International Statistical Classification of Diseases and Related Health Problems, 10th Revision* (ICD-10) details. Algorithms previously built for the analysis of these databases will be used to assess the patient outcomes [6, 38]. A complementary analysis will be performed using specific algorithms for SSI [39]. Specialists in charge of this analysis will be blinded to the randomisation arm. Secondary outcomes are the occurrence of POC including whole individual items of the composite criterion, the occurrence of SSI or any wound complication (superficial, deep, organ/space, or a problem of healing) up to 90 days following the procedure, the number of door openings, and the level of noise automatically collected by a network of sensors placed in the OR.

**Table 2:** Patient comorbidities and post-operative complications obtained from the French National Hospital Discharge Database and the *International Statistical Classification of Diseases and Related Health Problems, 10th Revision* (ICD-10)

Patient characteristics/comorbidities	Post-operative complication
Age	Surgical site infection
Gender	Thromboembolic complications
Public/private centre	Unplanned surgical revision
Elective/emergency	In-hospital mortality
Hypertension	Myocardial infarction
Ischemic heart disease	Cardiac failure
Cardiac arrhythmia	Stroke
Chronic heart failure	Renal failure/dialysis
Heart valve disease	Sepsis
Peripheral vascular disease	ICU admission
Dementia	Readmission within 72 h of discharge
Cerebrovascular disease	In-hospital mortality during readmission
Hemiplegia or paraplegia	
Chronic obstructive pulmonary disease	
Pulmonary circulation disorder	
Chronic respiratory failure	
Chronic alcohol abuse	
Cancer	
Cancer with metastasis	
Diabetes mellitus	
Obesity	
Chronic renal failure	
Pre-operative chronic dialysis	

### Study timeline

**Fig. 2:**
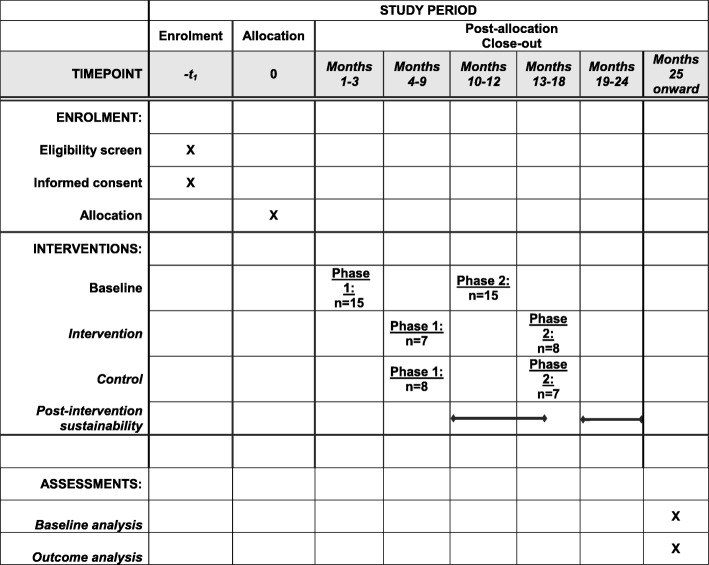
Trial schedule of enrolment, interventions, and assessments (as recommended by Standard Protocol Items: Recommendations for Interventional Trials (SPIRIT) Additional file 1)

The study will start with a baseline period of 3 months during which data on door opening and noise levels will be collected in the 30 ORs included (Fig. 2). The intervention period will last 6 months per centre. The centres will be included in two successive phases: a first phase including 15 centres (7 in the intervention arm and 8 in the control arm) and the 15 remaining centres in a second phase (8 in the intervention arm and 7 in the control arm). These two phases will be separated by a baseline period of 3 months (Fig. 2). This choice has been made for logistic reasons, to ease the installation of monitoring systems and the implementation of the intervention in a smaller group of centres. A post-intervention period of 6 months, keeping the monitoring system in place in the 30 ORs, will be used to assess the sustainability of the intervention. Due to the delay in the coding process of hospital discharge data and ICD-10 codes, we will analyse the outcomes at the end of our trial only.

### Sample size

This multicentre interventional study will include 30 healthcare facilities (HCFs) that represent 65 ORs for orthopaedic surgery. We made the hypothesis of a difference of POC rates of 7% vs 10%. Considering power of 80% (alpha = 5% bilaterally), we would need to include 2706 patients if the randomisation was individual. Because this trial is cluster randomised, an inflation factor has to be applied to this sample size, taking into account the correlation between patients clustered within a common cluster. In our design, patients are embedded with surgeons who are embedded in orthopaedic centres, which are randomly allocated. For the sample size calculation, we considered clustering at the surgeon level only, based on the assumption that the inter-patient correlation is stronger at the surgeon level than at the hospital level. Thus, considering the surgeon as the unit of clustering with an intraclass correlation coefficient of 0.05 and an average number of 54.5 patients per surgeon for a 6-month period, we calculated that 183 surgeons will be required, for an overall population of 9945 patients. Therefore, randomising 30 centres each accounting for an average of 6 surgeons allows the inclusion of 9945 patients to provide the nominal power to our trial. Although only the surgeon level correlation was taken into account in the sample size calculation, the statistical analysis will take into account the more complex hierarchical structure of the design.

### Recruitment

We will recruit a convenience sample of centres within our network of infection control specialists, quality officers and surgeons (western France and Paris area). An introductory email to contacts will be sent by the principal investigator, inviting them and their institution to consider participating. We will then arrange a meeting with surgeons, anaesthetists, managers and chief executives from interested sites to introduce our study and obtain written agreement to participate.

### Assignment of interventions

#### Sequence generation (i.e. randomisation)

Among the 15 centres included in the first study phase, 7 centres (clusters) will be randomly allocated to the intervention arm. During the second phase, 8 centres among the 15 remaining will be randomly allocated to the intervention arm (Additional file 1). A statistician, blinded to cluster identity and not involved in delivering the intervention, will generate the allocation sequence using computer-generated random numbers. The allocation sequence will be generated, including the hospital type (university hospitals, general hospitals and private hospitals) as a factor for stratification. This stratification is motivated by differences in organisation, staffing and patient recruitment across these hospital categories.

**Fig. 3:**
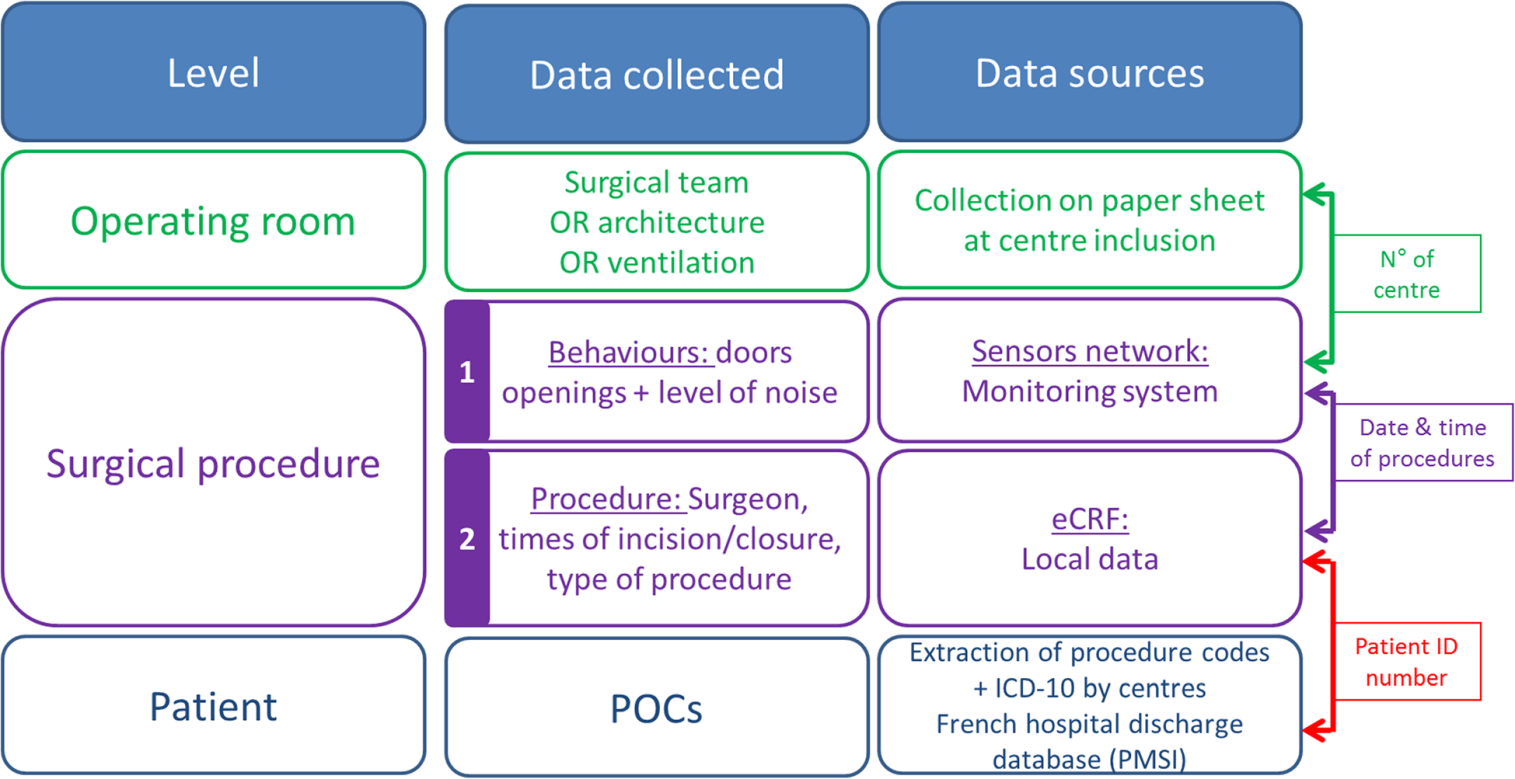
Description of data collected, data sources and key variables for the merging according to levels. OR, operating room; eCRF, electronic case report form; ICD-10*, International Statistical Classification of Diseases and Related Health Problems, 10th Revision*; POC, post-operative complication

#### Blinding

Surgical staff will be aware of the intervention they provide during the intervention period (i.e. they are not blinded). The statistician performing the analysis will be blinded to the identification of the sites. In both the intervention and the control conditions, the patients will be informed through a letter that the site is participating in a research study. Medical doctors specialising in the analysis of data from the local and national hospital discharge database of diagnosis and procedure codes will be blinded to the randomisation arm.

### Data collection and management

Information will be collected at three different levels. At the centre level, we will collect data on randomised hospitals (status and surgical activity) and their surgical environment characteristics (air changes of filtered air per hour, positive pressure, temperature, relative humidity, particle contamination class, kinetics of particle decontamination class). Characteristics of senior surgeons performing hip/knee replacements will be recorded (age, gender, function, experience in the function, role in the surgical procedure). At the patient level, variables on intra-operative staff behaviour (frequency of door opening and noise levels), the patients and surgical procedures (patient ID number, type of procedure, date, incision time, wound closure time and the OR ID) will be collected. These data will be collected by the network of wireless sensors described above and by extraction from OR information systems.

A list of each patient’s comorbidities and crude post-operative events will be extracted from the local and the national hospital discharge database (PMSI) and the ICD-10 (Table 1 and Fig. 3). The primary outcome of the study, as defined above, will be identified using algorithms previously developed for the PMSI and ICD-10 databases to assess patients’ outcomes after hip replacement [6, 38]. A complementary assessment will be performed using specific algorithms for SSI [39]. This algorithm previously developed for detecting hip or knee arthroplasty-related infections, showed a sensitivity (Se) of 98% (97.1–98.9) and a specificity (Spe) of 71 (67.8–73.4), a positive predictive value (PPV) of 63% (59.8–65.8) and negative predictive value (NPV) of 99% (98.1–99.5). Using a more specific case definition, based on a sample of 681 hospital stays, Se was 97%, Spe was 95%, PPV was 87%, and NPV was 98% [39].

### Statistical methods

#### Data analysis

The results from the statistical analyses will be reported according to the Consolidated Standards of Reporting Trials (CONSORT) statement recommendations and its extensions for cluster randomised trials and for non-pharmacological interventions. No interim analysis will be performed. All eligible patients will be taken into account according to the initial allocation. OR, surgeon and patient characteristics will be reported using descriptive statistics. The primary outcome will be analysed using a logistic mixed model taking into account the stratification factor as a fixed effect [40]. Patients will be the unit of analysis and will be considered embedded with surgeons who will themselves be considered embedded in orthopaedic centres. Adjusted analyses may also be performed, notably in the case of baseline imbalance, a situation often encountered in cluster randomised trials, notably due to a limited number of randomisation units. A sensitivity analysis may also be planned depending on the results observed as regard to the adherence to the intervention. Intraclass correlation will be estimated within each group.

For secondary outcomes, components of the primary outcome will be analysed with an equivalent method as for the main criteria. The frequency of doors opening will be analysed with a mixed effect Poisson regression model with the duration of procedure as offset. The duration of hospital stay and the surgical intervention duration (from wound incision to closure) will be analysed using a mixed linear model after application of a Box-Cox transformation if necessary.

#### Data monitoring, harms and auditing

We do not have a data safety and monitoring board because our trial poses minimal risk. We do not plan to conduct any interim analyses for our primary outcome. Any unintended effect of the trial intervention will be tracked by weekly calls with the participating sites. The Nantes University Hospital, Sponsor of the study, provides insurance in case of negligent harm. We will not conduct any audits of trial conduct. Independent scientific experts will be part of the study scientific committee to provide external advice on the conduct and reporting of the study.

### Ethics

#### Consent, confidentiality and access to data

We will follow the Ottawa Statement on the ethical design and conduct of cluster randomised trials [41]. Our intervention will be delivered at the cluster level (operating theatre) and we were approved for an alteration of the individual patient and staff consent. The Tri-council Policy Statement on the Ethics for Research Involving Human Participants indicates that an alteration of individual informed consent is permitted when the research involves minimal to no risk to participants [42]. The alteration is unlikely to adversely affect the welfare of participants, and it is impracticable to carry out the research given the cluster-level intervention and research design if the prior consent of patients and staff (including surgeons) is required. We obtained a waiver of consent from our Research Ethics Board (Nantes University Hospital, Groupe Nantais d’Ethique dans le Domaine de la Santé) for data collection because we are not obtaining any identifiable private information from patients and staff. Participating patients will be informed by the surgeon or the anesthetist in charge before the surgical procedure, using an information letter validated by our Research Ethics Board. OR staff will be informed by an information note and enhanced communication.

Data on the surgical environment (OR characteristics and surgeons) will be collected on a paper form. A code will be attributed to surgeons in each centre. The link between the code and the surgeon identity will be kept by the investigators. These documents will be stored in a closed cabinet.

The network of sensors used for the monitoring of door opening and the noise levels collect aggregated data without the possibility of identifying individuals. These data will be stored on a dedicated secure server. Data on surgical procedures (patient ID number, type of procedure, date, incision time, wound closure time and the OR ID) extracted from OR information systems will be downloaded onto a dedicated server (Hestia) housed at the Nantes University Hospital, providing automatic encryption. Behavioural data and data on procedures will be merged on this server with the date and time of the procedure as a key variable (Fig. 3).

Hospital discharge and ICD-10 databases will be quarterly extracted from centres and stored on a dedicated server (Hestia) housed at the Nantes University Hospital, after automatic encryption of the patient ID number. The merging of these databases with the procedures and behavioral databases will be performed using the encrypted patient ID number as the key variable (Fig. 3). Finally, the encoded, linked, national administrative healthcare databases will be stored on the dedicated server housed at the Nantes University Hospital (see the “Data collection” section).

We received ethics approval from the “Nantes University Hospital, Groupe Nantais d’Ethique dans le Domaine de la Santé” to conduct the intervention. We obtained ethics approval from the “Comité National Informatique et Liberté” to obtain encoded baseline and outcome data on patients. “Comité National Informatique et Liberté” provides approval for research projects using the collection, storage, and use of hospital databases.

### Dissemination policy, authorship eligibility and data sharing plans

We aim to publish our findings regardless of negative or null results. All named authors in the protocol will be offered participation in the paper on final outcomes and any subsequent papers. We will not use any professional writing services. Due to institutional policies, we will not be able to grant public access to the participant-level dataset. We may be able to provide a statistical code. We will grant public access to all intervention materials.

### Trial status

This article refers to the protocol version 2 dated 6 November 2018. The recruitment will begin on 2 January 2019, with completion of recruitment expected in February 2020.

## Discussion

In the present protocol, we suggest the implementation of a cluster randomised trial to assess the impact of a bundle of measures based on the monitoring and feedback of intra-operative behaviour combined with an adaptive approach for their optimization, to prevent POC. We will adopt key points of behavioural change theories providing goal-setting, feedback and action planning in changing surgical staff behaviour in the OR during procedures [43]. The monitoring system, with connected wireless sensors developed and designed for the study, will provide staff with feedback on their behaviour and represent the corner stone of the intervention. Added measures will take inspiration from successful experience in the field [23]. The CUSP used in different areas for improvement in the quality of care, including surgery, will also represent a source of inspiration [44]. This strategy will help in the design of adaptive work, managing the staff’s attitudes and values, and engaging senior executives in patient safety initiatives [45].

Orthopaedic surgery involving knee and hip arthroplasty has been included according to the following criteria: a cutaneous approach, clean contamination class (Altemeier’s class I) and the frequency and the reproducibility of the procedure. An additional argument for this choice is the strong concern and awareness of orthopaedic surgeons about the risk of POC, especially infections. However, the methodological disadvantage of orthopaedic surgery is the low incidence of POCs. In consequence, the number of procedures to include is relatively high with an expected number of 9945 procedures in total. To ease the implementation of the protocol, data will be passively collected. All comorbidities and POCs will be identified using the local and national hospital discharge database, giving the possibility to follow patients 30 and 90 days after the procedure in all HCFs. Previous studies have demonstrated the feasibility and applicability of this method [6, 46]. One main limitation of this strategy is the potential misclassification of outcomes. Underestimation of their frequency remains possible; however, this would be expected to be similar in both groups. Nevertheless, we cannot completely exclude differential misclassification between the intervention and control groups.

We chose to select two behaviour surrogates for this study: the frequency of door opening and the level of noise. This selection was performed in a spirit of simplifying messages and data collection. Doors opening may be considered a surrogate of the organisation and discipline in the OR while the level of noise is a surrogate of the atmosphere and communication. Both are essential for effective conduct of the surgery. However, this has to be balanced so as not to fall into the excess of entries/exits and interruption/distraction, with potential consequences for the contamination of the environment and the performance of the surgical team.

Expected outputs from this study will be, first to build interventions based on an innovative tool to optimise staff awareness and behaviour according to the context and constraints of surgical activities. Second, this will also potentially demonstrate, using an accurate method, the impact of intra-operative behaviour on patient outcomes. Third, this will allow establishment of a standard of behavioural best practice in the OR. Finally, it will provide a broad evaluation of practices across a large panel of orthopaedic centres with an assessment of the benchmarking process. A reduction, even modest, in POC rates may have important consequences in term of public health, and will probably lead to the evolution of perception and measures for the control of intra-operative risk. The field of discipline in the OR appears poorly explored and seems to represent an interesting and promising way to improve surgical performance in the future.

This challenging project gathers specialists from several disciplines (infection control, epidemiology, surgery, anaesthesiology, psychology and engineering) and will confirm or disprove the impact of intra-operative staff behaviour on the occurrence of POC. Even if applied in orthopaedic surgery and knee/hip replacement, the findings may be transposable to other surgical specialties and procedures with potentially broad application of the monitoring/feedback system and adaptive approach for the optimisation of intra-operative behaviour.

## Additional file

Additional file 1: SPIRIT Checklist. (DOC 98 kb)

## Abbreviations

CUSP: Comprehensive Unit-based Safety Programme
dB: Decibels
GEE: Generalized estimation equation
HCFs: Healthcare facilities
ICD-10: *International Statistical Classification of Diseases and Related Health Problems, 10th Revision*
ID: Identification
OR: Operating room
PDCA: Plan-Do-Check-Act
POC: Post-operative complications
SPIRIT: Standard Protocol Items Recommendations for Interventional Trials
SSI: Surgical site infections
TIDieR: Template for Intervention Description and Replication

## Authors’ information

GB is an infection control specialist who performed his PhD under the supervision of JCL, on the intra-operative behaviour and the consequences for risk of infection.
